# Design of Research on Performance of a New Iridium Coordination Compound for the Detection of Hg^2+^

**DOI:** 10.3390/ijerph14101232

**Published:** 2017-10-16

**Authors:** Hailing Ma, Sang-Bing Tsai

**Affiliations:** 1 Zhongshan Institute, University of Electronic Science and Technology of China, Zhongshan 52800, China; 2 Economics and Management College, Civil Aviation University of China, Tianjin 300300, China; 3 School of Science, Jinggangshan University, Ji’an 343009, China; 4 College of Chemical Engineering and Biological Engineering, Zhejiang University, Hangzhou 310000, China

**Keywords:** heavy metal, iridium coordination compound, new auxiliary ligands, quantum efficiency, sustainability, green environment

## Abstract

Heavy metal pollution has become one of the most significant pollution problems encountered by our country in terms of environment protection. In addition to the significant effects of heavy metals on the human body and other organisms through water, food chain enrichment and other routes, heavy metals involved in daily necessities beyond the level limit could also affect people’s lives, so the detection of heavy metals is extremely important. Ir (III) coordination compound, considered to be one of the best phosphorescent sensing materials, is characterized by high luminous efficiency, easy modification of the ligand and so on, and it has potential applications in the field of heavy metal detection. This project aims to product a new Ir (III) functional coordination compound by designing a new auxiliary ligand and a main ligand with a sulfur identification unit, in order to systematically investigate the application of iridium coordination compound in the detection of the heavy metal Hg^2+^. With the introduction of the sulfur identification unit, selective sensing of Hg^2+^ could be achieved. Additionally, a new auxiliary ligand is also introduced to produce a functional iridium coordination compound with high quantum efficiency, and to diversify the application of iridium coordination compound in this field.

## 1. Introduction

In recent years, the pollution of sea water has become increasingly severe in China, especially heavy metal pollution, which has become one of the major pollution problems encountered by our country in water environment protection field. Heavy metals in water are non-biodegradable; therefore, even though their contents are very low, they may be condensed after continuous concentration through the food chain level by level, leading to serious damage to the organisms at the top of the food chain. This means adverse effects on human health, as well as outbreaks of publicly harmful diseases. The famous diseases “minamata” and “itaiitai” in Japan in the 1950s or 1960s, for example, were caused by mercury and cadmium, respectively. Heavy metal pollution can produce various diseases, even cancer, the harm of which may also be passed on to the next generation. In recent years, since heavy metal pollution incidents have been frequent, reports on the “cancer village” caused by heavy metal pollution have also been seen in newspapers from time to time. Therefore, the heavy metal pollution has received great attention from the government. In the Twelfth Five-Year Plan, it is clearly indicated that the integrated control of heavy metal pollution shall be enhanced, focusing on the control of five types of heavy metals, i.e., lead, mercury, cadmium, chromium and arsenic. It also indicates that the improvement of capacities for environmental monitoring, warning and emergency response shall be consolidated. Recently, it has been reported by a journalist that the mercury level in some of the cosmetics in China is 60,000 times higher than the normal level and that cosmetics in which the mercury level exceeds the limit have led to nephrotic syndrome in a female consumer. Heavy metal pollution is no longer confined to industrial production and actually affects people’s daily lives. This means there will be a great demand for the monitoring and detection of heavy metal pollution in the coming period [1,2,3,4,5,6,7,8,9,10,11,12,13,14,15,16,17,18,19,20,21,22,23,24,25,26,27,28,29,30].

Iridium coordination compound is a kind of phosphorescent coordination compound that has been researched more frequently than all coordination compounds for other metals. Since the atomic number of iridium is relatively large, the coordination compound can produce strong spin-orbit coupling, which is helpful for phosphorescence emission. Splitting of orbit d in the iridium metal ion is large, so it could avoid the reduction of phosphorescence emission efficiency caused by the interaction with the metal-ligand charge transfer (MLCT) state of the coordination compound. Trivalent ions of iridium could form very stable neutral and ionic molecules together with ligands. Additionally, iridium coordination compound is also characterized with other advantages, such as long excited state, high luminous efficiency and easy adjustment of luminous color. These properties make iridium coordination compound greatly advantageous in the manufacture of multi-signal responsive phosphorescent chemical sensors. An identification unit capable of identifying heavy metal cations, such as a sulfur atom, is introduced into the Ir (III) coordination compound, through the design of the molecular structure. The excited state properties of the coordination compound could be changed through complexation between the accepting unit and the heavy metal ion to be detected, leading to a change in absorption or emission or in the electrochemical properties, thus to realize the detection of such a cation [31,32,33,34,35,36,37,38,39,40,41,42,43,44,45,46,47,48,49,50].

Since the oxadiazole compound is characterized with good thermal stability and hydrolytic stability, as well as high electron affinity and strong blue fluorescence, it could be used as a typical luminescent material. In particular, the 1,3,4-oxadiazole compound with the 2,5-diaryl replaced is a good electron acceptor that can be introduced into the luminescent material to enhance the ability to accept electrons, i.e., to reduce the molecular HOMO energy level, thus to make the complex produce a strong photoluminescence [51,52,53,54,55,56,57,58].

The research in this paper starts with the design of the new auxiliary ligand, 2-(5-phenyl-1,3,4-oxadiazole-2-radical)-phenol, and its derivatives, as well as the design of the main ligand, benzothiophene-2-pyridine, to synthesize two kinds of new neutral Ir (III) coordination compounds, followed by research on their luminescent properties and the detection capabilities for heavy metal Hg^2+^. In the molecular design of these two coordination compounds, the sulfur atom is introduced therein, which, as a soft base, could react with the soft acid Hg^2+^, thus to realize the detection of Hg^2+^. The result of research will provide the theoretical and technical support for further development and ultimate commercial application of the phosphorescent chemical sensor [59,60,61,62].

## 2. Implementation Plan

### 2.1. Experimental Instruments

The NMR spectrum was determined by the Bruker BioSpin GMBH nuclear magnetic resonance instrument (400 MHz, Bruker, Switzerland, internal standard with TMS). The UV-Vis absorption spectrum was determined by TU-1900 UV-Vis spectrophotometer (Beijing General Analytical Instrument Co., Beijing, China), and X-ray single crystal diffraction was measured by X-ray single crystal diffractometer (manufactured by Brooke Corporation, Saarbruecken, Germany).

### 2.2. Synthesis and Characterization of the Main Ligand Benzothiophene-2-pyridine (BTP)

The main ligand is produced through reaction between benzothiophene-2-boric acid and 2-bromopyridine in the solution of tetrahydrofuran and H_2_O in which the anhydrous sodium carbonate is added as the alkali source, catalyzed by tetrakis (triphenylphosphine) palladium, and purified via column chromatography separation. Productivity: 77.58%. ^1^H NMR: (300 MHz, CDCl3) δ 8.63 (d, *J* = 4.4 Hz, 1H), 7.93–7.76 (m, 4H), 7.71 (td, *J* = 7.8, 1.5 Hz, 1H), 7.41–7.30 (m, 2H), 7.20 (t, *J* = 9.0 Hz, 1H).

### 2.3. Synthesis of a New Type of Auxiliary Ligand

**Figure 1:**
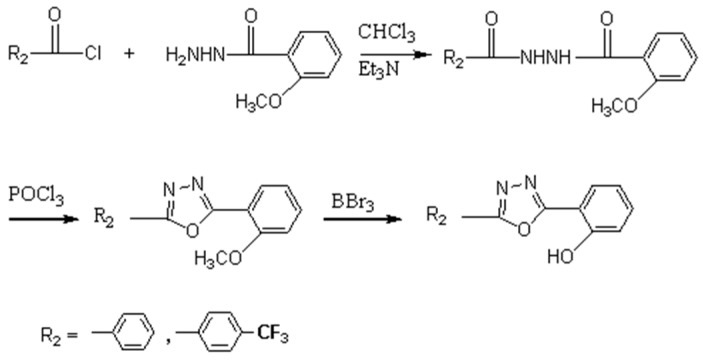
Synthesis routes of 2-(5-phenyl-2-(-1,3,4)-two-radical)-phenol-auxiliary ligands.

Figure 1 shows the synthesis flow of the auxiliary ligand, 2-(5-phenyl-1,3,4 oxadiazole-2-radical) phenol. Firstly, benzoyl chloride reacts with 2-methoxy-benzohydrazide to get precipitate under the effect of triethylamine. Secondly, the precipitate undergoes the ring formation step under the effect of POCl_3_, to generate the ligand that contains 1,3,4-oxadiazole radical. Finally, the ligand is transformed to the target auxiliary ligand under the effect of BBr3, taking demethoxy as the hydroxyl.

### 2.4. Synthesis and Characterization of the Coordination Compound

**Figure 2:**
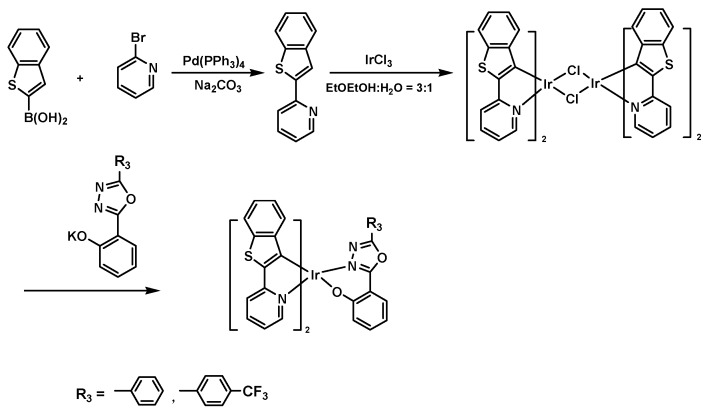
Synthesis route of Ir (III) coordination compound.

Figure 2 shows the molecular structure and synthesis flow of Ir (III) coordination compound. The neutral iridium coordination compound is synthesized from IrCl3 in two steps. In the first step, the main ligand and IrCl_3_ react in the solution of 2-ethoxyethanol and H_2_O, to form the di-chlorendic intermediate. In the second step, coordination between the di-chlorendic intermediate and the auxiliary ligand is established to form the neutral Ir (III) coordination compound.

### 2.5. Characterization of Coordination Compound

Ir1 coordination compound yield: 54.71%. ^1^H NMR (300 MHz, DMSO-*d*_6_) δ 8.65 (d, *J* = 6.0 Hz, 1H), 8.47 (d, *J* = 5.3 Hz, 1H), 7.99–7.88 (m, 1H), 7.87–7.76 (m, 3H), 7.76–7.70 (m, 1H), 7.59–7.46 (m, 2H), 7.26–7.16 (m, 1H), 7.16–7.04 (m, 2H), 6.82 (t, *J* = 7.6 Hz, 1H), 6.61 (dd, *J* = 8.7, 0.8 Hz, 1H), 6.51 (ddd, *J* = 8.0, 6.9, 1.1 Hz, 1H), 6.13 (d, *J* = 7.8 Hz, 1H), 6.02 (d, *J* = 8.0 Hz, 1H). Element analysis theory: C 56.52, H 2.96, N 6.59; Measured: C 53.6, H 2.7, N 6.28.

**Figure 3:**
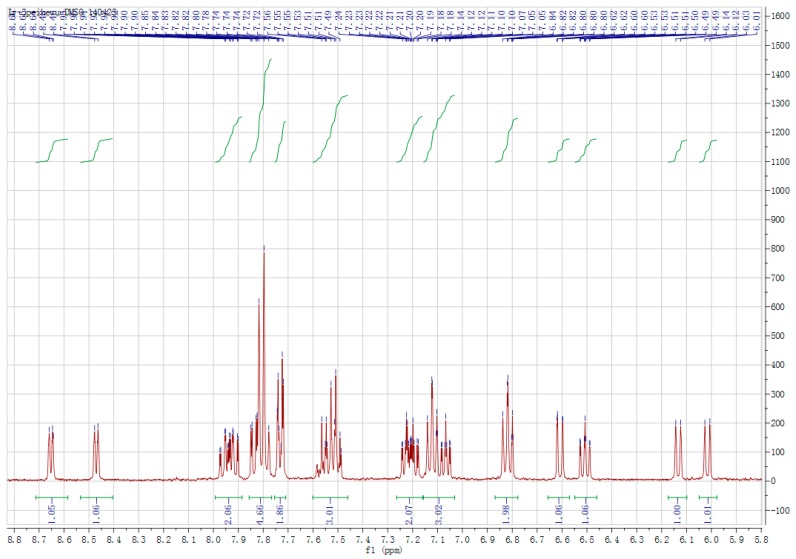
^1^H NMR spectra of coordination compound Ir1.

Figure 3 is the NMR image of the Ir1 coordination compound. The recorded hydrogen spectrum is a single-pulse experiment, that is, after a pulse action, the sampling is started. In order to make the obtained spectrum have a good signal to noise ratio, it is needed to accumulate the test, that is, repeat the process. Ir1 coordination compoundes Productivity: 54.71%. ^1^H NMR (300 MHz, DMSO-*d*_6_) δ 8.65 (d, *J* = 6.0 Hz, 1H), 8.47 (d, *J* = 5.3 Hz, 1H), 7.99–7.88 (m, 1H), 7.87–7.76 (m, 3H), 7.76–7.70 (m, 1H), 7.59–7.46 (m, 2H), 7.26–7.16 (m, 1H), 7.16–7.04 (m, 2H), 6.82 (t, *J* = 7.6 Hz, 1H), 6.61 (dd, *J* = 8.7, 0.8 Hz, 1H), 6.51 (ddd, *J* = 8.0, 6.9, 1.1 Hz, 1H), 6.13 (d, *J* = 7.8 Hz, 1H), 6.02 (d, *J* = 8.0 Hz, 1H). Theoretical value of element analysis: C 56.52, H 2.96, N 6.59; actual measurement: C 53.67, H 2.7, N 6.28.

**Figure 4:**
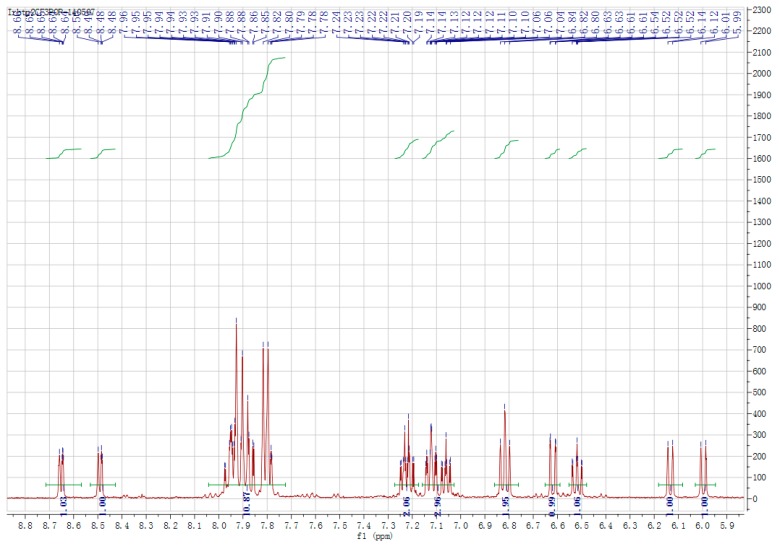
NMR spectra of coordination compound Ir2.

Figure 4 is the NMR image of the Ir2 coordination compound. Ir2 coordination compounds Productivity: 53.38%. ^1^H NMR (300 MHz, DMSO-*d*_6_) δ 8.65 (d, *J* = 6.0 Hz, 1H), 8.49 (dd, *J* = 3.8, 2.9 Hz, 1H), 8.00–7.84 (m, 7H), 7.83–7.77 (m, 4H), 7.27–7.18 (m, 2H), 7.15–7.03 (m, 3H), 6.82 (t, *J* = 7.6 Hz, 2H), 6.62 (dd, *J* = 8.7, 0.8 Hz, 1H), 6.52 (ddd, *J* = 8.0, 6.9, 1.1 Hz, 1H), 6.13 (d, *J* = 7.9 Hz, 1H), 6.00 (d, *J* = 7.9 Hz, 1H). Theoretical value of element analysis: C 53.64, H 2.64, N 6.10; actual measurement: C 53.41, H 2.80, N 6.25.

### 2.6. Study on the Performance of 2.5 Iridium Coordination Compound in Detection of Hg^2+^

Test the performance of iridium coordination compound in detection of Hg^2+^ according to the color change when Hg^2+^ is put into the different kinds of solutions of iridium coordination compounds.

## 3. Experimental Results and Discussion

### 3.1. Crystal Structure

**Figure 5:**
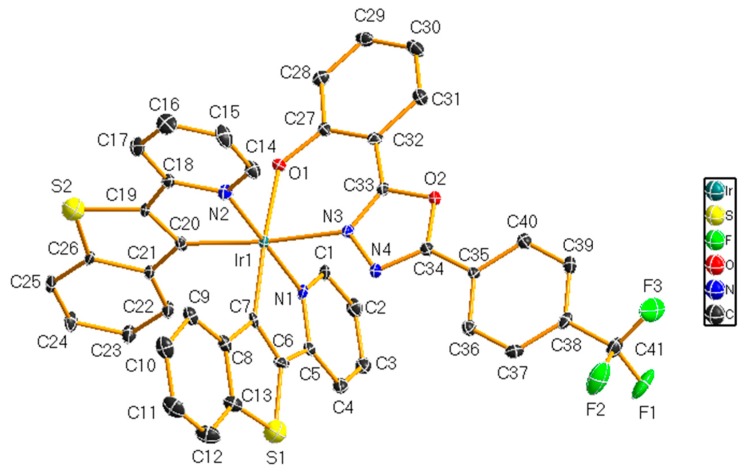
X-ray single crystal diffraction pattern of coordination compound Ir2 crystal structure.

The compound Ir2 will volatilize in the mixture of dichloromethane and methyl alcohol to get crystal, which is obtained from X-ray single crystal diffraction. Before testing, it is necessary to glue the selected crystal sample with the capillary glass, and then insert the colored sample and the capillary glass into a specially made small copper column and put it into the sample rack for testing. The shape of the crystal is ellipsoid, as shown in Figure 5. Bond lengths and angles are listed in Table 1.

### 3.2. UV Vis Absorption Spectrum

**Figure 6:**
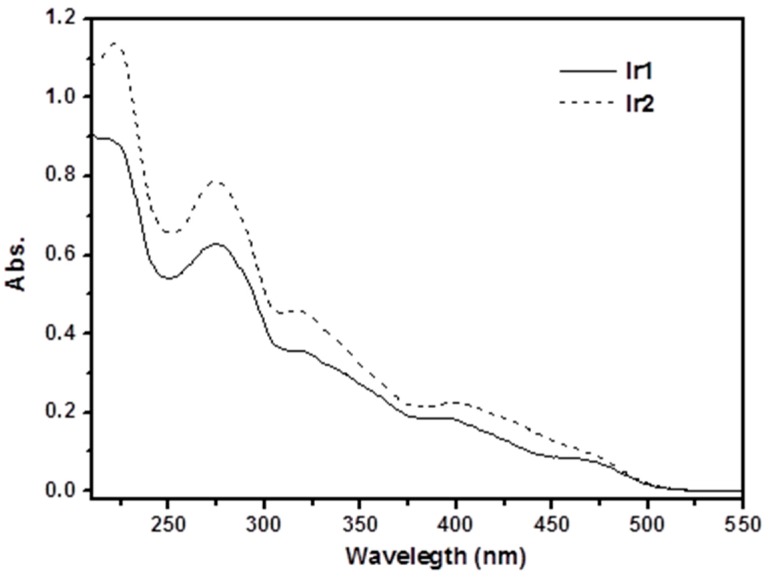
UV Vis absorption spectra of coordination compound Ir1, Ir2 acetonitrile solution (2.0 × 10^–5^ mol/L).

The process was to turn on the power, turn on the UV-visible spectrophotometer on the switch, turn on the computer software UV probe, let the self-test function operate for about 5 min, and set the instrument-related parameters. The wavelength range was set to 550–200 nm, high-speed detection. Figure 6 shows the ultraviolet-visible absorption spectrum of iridium coordination compounds Ir1 and Ir2 in the acetonitrile solution with a concentration of 2.0 × 10^–5^ mol/L at room temperature, and data on absorption peak of two coordination compounds are listed in Table 2. As shown in the figure, the coordination compounds Ir1 and Ir2 are similar in terms of the shape of absorption spectrum, but differ slightly in the intensity. In the range of 210–375 nm, both coordination compound Ir1 and Ir2 have a high absorption peak. The molar absorption coefficient is within a range of 4.2 to 56.9 × 103 M-1 cm^−1^, in which the absorption peak is attributable to the spin-allowable π→π* transition that takes the ligand as the center. The absorption peak in the low energy range (>375 nm) could be attributable to the metal-ligand charge transition, i.e., MLCT absorption, including the combined transition absorption of spin-allowable MLCT and spin-prohibited MLCT.

### 3.3. Phosphorescent Emission Spectrum

**Figure 7:**
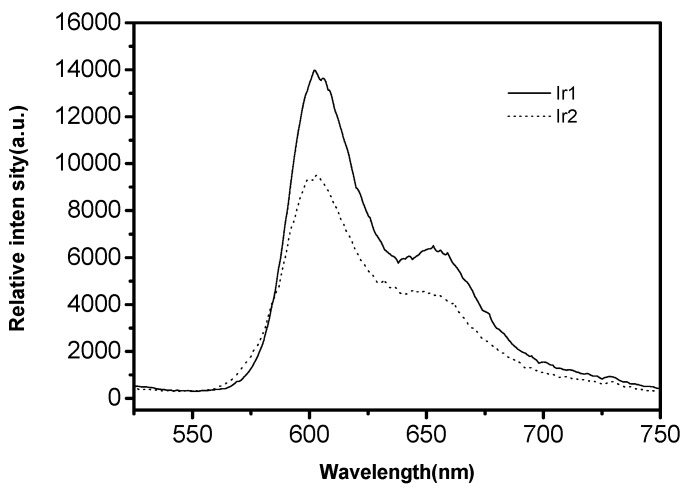
Acetonitrile coordination compoundes of Ir1 and Ir2 (2 × 10^−5^ mol/L) phosphorescence emission spectra.

Figure 7 shows the phosphorescent emission spectrum of the coordination compounds Ir1 and Ir2 in the acetonitrile solution (2.0 × 10^−5^ mol/L) at the room temperature, and the data on the phosphorescent emission peak are listed in Table 1. When the coordination compounds Ir1 and Ir2 are excited at 365 nm, strong MLCT phosphorescent emission could be formed, with the emission peak at 603 nm and 655 nm respectively, and the color emitted by the coordination compounds is red. As it is shown in the emission spectrum, the coordination compounds Ir1 and Ir2 are similar in terms of the shape of emission spectrum, but differ slightly in intensity.

### 3.4. Detection Performance of Ir Coordination Compoundes on Mercury Ions

**Figure 8:**
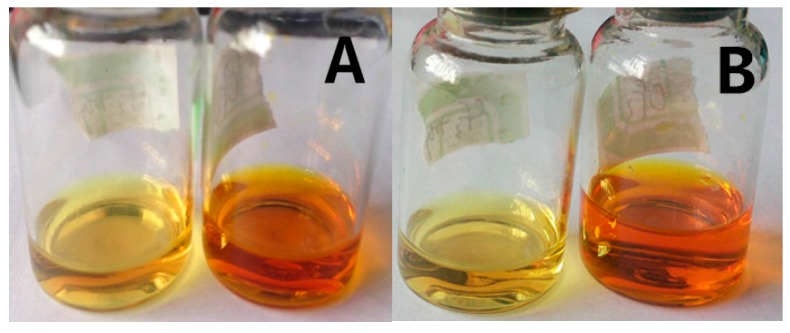
By adding 3 equivalents of metal ions with matter IR1 (**A**), the color change photos of Ir2 (**B**) acetonitrile solution (2.0 × 10^−5^ mol/L) in acetonitrile. (The two figures on the left side show the solution of the coordination compounds with the addition of Hg2^+^ color, the figures on the right, coordination compound color).

Figure 8 shows that the color of two coordination compound solutions changes significantly within 1 min after Hg^2+^ is added. Upon addition of Hg^2+^, the color of the coordination compounds Ir1 and Ir2 changes from orange red to light yellow, suggesting that both coordination compounds could be used to identify Hg^2+^ by means of visual observation.

### 3.5. Ion Selective and Competitive Experiment

A chemical sensor with good performance should have high selectivity. Cd^2+^, Pb^2+^, Ag^+^, Fe^2+^, Co^2+^, Ni^2+^, Zn^2+^, Mg^2+^, K^+^ and Na^+^ can effectively quench the phosphorescence of two quantum dots. There is a difference in the phosphorescence of the Hg^2+^for the two quantum dots.

**Figure 9:**
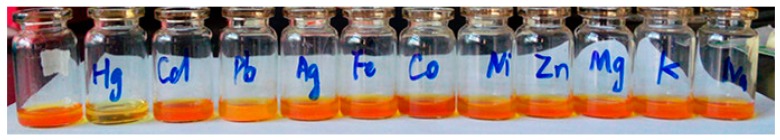
The response of coordination compound Ir1 acetonitrile solution to various metal ions (Hg^2+^, Cd^2+^, Pb^2+^, Ag^+^, Fe^2+^, Co^2+^, Ni^2+^, Zn^2+^, Mg^2+^, Na^+^ and K^+^).

We have reviewed the experiment in which the selectivity of coordination compounds Ir1 and Ir2 to 11 metal ions are researched, including Hg^2+^, Cd^2+^, Pb^2+^, Ag^+^, Fe2^+^, Co^2+^, Ni^2+^, Zn^2+^, Mg^2+^, K^+^ and Na^+^. As shown in Figure 9, when 3 equivalents of Hg^2+^ are added into the solution of coordination compound Ir1, the color of solution changes from orange red to light yellow within 1 min, but when 3 equivalents of any other ion are added, such as Cd^2+^, Pb^2+^, Ag^+^, Fe^2+^, Co^2+^, Ni^2+^, Zn^2+^, Mg^2+^, K^+^ and Na^+^, the color of solution sees no significant change. The selectivity experiment of coordination compound Ir2 has got the same results as Ir1, except that the color change is not so obvious. Therefore, both coordination compounds synthesized in the experiment, i.e., Ir1 and Ir2, have a certain selectivity to Hg^2+^, of which the selectivity of Ir1 is higher.

## 4. Innovation

With design and synthesis of the oxadiazole-contained auxiliary ligand, as well as the new iridium coordination compound with the main ligand that contains a sulfur accepting unit, selective sensing to the cation Hg^2+^ could be achieved, and that is a great innovation in the molecular structure design.

## 5. Conclusions

The detection method is characterized ny short response time and easy operation, and does not rely on large instruments and equipment, which could reduce the detection costs. The method is applicable to the detection of heavy metal ions in bodies of water.

## Figures and Tables

**Table 1:** Bond lengthes (nm)-S, bond angles (°C)-T.

Parameter	S	T
Ir-C20	0.2009	0.2032
Ir-N8	0.1988	0.1971
Ir-O1	0.2167	0.2169
Ir-N3	0.2036	0.2010
Ir-N1	0.2072	0.2070
Ir-C7	0.1996	0.2003

**Table 2:** UV-Vis absorption spectra and phosphorescence emission spectra of coordination compounds Ir1 and Ir2.

Coordination Compound	UV Absorption [*λ*, nm (*ε*, 10^3^ M^−1^ cm^−1^)]	Phosphorescence Emission (*λ*_max_, nm)
Ir1	218 (44.9), 274 (31.5), 318 (17.9), 394 (9.4), 461 (4.2)	603/655
Ir2	221 (56.9), 274 (39.5), 318 (23.0), 399 (11.3), 462 (5.4)	603/655
